# Advances in Fungal Infection Research: From Novel Diagnostics to Innovative Therapeutics

**DOI:** 10.3390/jof11100693

**Published:** 2025-09-25

**Authors:** Célia Fortuna Rodrigues, Lucia Černáková

**Affiliations:** 1 Associate Laboratory i4HB—Institute for Health and Bioeconomy, University Institute of Health Sciences—CESPU (IUCS-CESPU), 4585-116 Gandra, Portugal; 2 UCIBIO—Applied Molecular Biosciences Unit, Translational Toxicology Research Laboratory, University Institute of Health Sciences (1H-TOXRUN, IUCS-CESPU), 4585-116 Gandra, Portugal; 3 LEPABE—Laboratory for Process Engineering, Environment, Biotechnology and Energy, ALiCE-Associate Laboratory in Chemical Engineering, Faculty of Engineering, University of Porto, Rua Dr. Roberto Frias, 4200-465 Porto, Portugal; 4 Department of Microbiology and Virology, Faculty of Natural Sciences, Comenius University in Bratislava, Mlynská dolina, Ilkovičova 6, 842 15 Bratislava, Slovakia; lucia.cernakova@uniba.sk

Invasive and superficial fungal infections continue to impose a significant global health burden, with rising morbidity and mortality rates particularly affecting immunocompromised populations [1,2]. The emergence of antifungal resistance (AFR), coupled with the rise in the prevalence of opportunistic fungal pathogens, underscores the urgent need for innovative diagnostic approaches and alternative therapeutic strategies [3,4]. Indeed, fungal infections represent one of the most complex challenges in modern medicine, affecting millions of patients worldwide [5,6]. The clinical spectrum ranges from superficial mucocutaneous infections to life-threatening invasive diseases, with opportunistic pathogens such as *Candida*, *Aspergillus*, and *Pneumocystis* species leading the epidemiological burden [7,8], particularly in immunocompromised patients, including those undergoing chemotherapy, organ transplantation, or living with HIV/AIDS, who comprise an expanding population at risk for severe mycoses [9,10].

The diagnostic landscape for fungal infections has undergone significant transformation in recent decades [11,12]. Traditional culture-based methods, while remaining the gold standard, are often time-consuming and may lack sensitivity for certain pathogens [13]. The development of molecular diagnostic techniques, including real-time PCR assays and next-generation sequencing, has revolutionized pathogen detection and identification, including in fungal species [14,15]. These methods enable rapid, accurate diagnosis that can be decisive for patient outcomes, especially in critically ill populations where an early intervention is crucial [16,17].

While the antifungal therapeutic arsenal has expanded considerably, challenges persist [18,19]. The limited number of antifungal drug classes, combined with the emergence of AFR, poses major obstacles to effective treatments [20,21]. Azole resistance in *Aspergillus fumigatus*, echinocandin resistance in *Candida* species, and the global spread of multidrug-resistant *Candida auris* exemplify the evolving threat landscape [22,23]. Novel therapeutic approaches are being explored across multiple fronts [24,25]. Structure-based drug design has yielded promising compounds targeting specific fungal pathways, while drug repurposing strategies have identified unexpected antifungal properties in existing medications [26,27]. Additionally, natural products and antimicrobial peptides represent other opportunities for therapeutic development, offering potential alternatives to conventional drugs [28,29].

The importance of systematic surveillance in healthcare settings cannot be overstated [30,31]. Effective monitoring programs enable early detection of outbreaks, facilitate antimicrobial stewardship, and guide infection prevention strategies [32]. The implementation of screening protocols for high-risk patients has proven valuable in predicting disease progression and optimizing therapeutic interventions [33,34]. Indeed, healthcare-associated fungal infections require comprehensive infection control measures [35]. The emergence of environmental pathogens and the challenges posed by biofilm formation (on medical devices) require multidimensional prevention policies [36,37]. Understanding the epidemiology of healthcare-associated mycoses is imperative for developing targeted interventions and reducing transmission risks [38].

Recent advances in understanding fungal pathogenesis have revealed the complexity of host–pathogen interactions [39,40]. The role of host immunity, particularly in immunocompromised patients, determines disease susceptibility and progression [41,42]. Fungal virulence factors, including morphological transitions, biofilm formation, and immune evasion mechanisms, contribute to pathogenic success [43,44]. In addition, the concept of polymicrobial infections has gained attention, with evidence suggesting that inter-microbial interactions can significantly influence disease outcomes [45,46]. These interactions may involve competitive or synergistic relationships between different fungal species or between fungi and bacteria, potentially affecting treatment strategies and patient prognosis [47].

Epidemiological studies continue to identify key risk factors for fungal infections across diverse populations [48,49]. Diabetes mellitus, advanced age, immunosuppressive therapy, and prolonged hospitalization represent well-established predisposing factors [50,51]. Understanding these risk profiles enables targeted screening and prevention strategies for high-risk populations [52]. Geographic variation in fungal disease burden reflects environmental factors, endemic species distribution, and healthcare infrastructure differences [53,54]. Regional studies offer basic insights for public health planning and resource allocation, particularly in areas with limited diagnostic capabilities or restricted access to antifungal therapy [55,56].

The integration of artificial intelligence and machine learning into diagnostic platforms promises to enhance disease detection and prediction capabilities [57,58]. Advanced imaging techniques, biomarker discovery, and point-of-care testing exemplify emerging frontiers in fungal diagnostics [59,60]. Precision medicine approaches, incorporating host genetic factors, pathogen characteristics, and environmental variables, may enable individualized treatment strategies [61,62]. The development of immunotherapeutic approaches, including vaccine strategies and immune modulators, offers additional therapeutic possibilities [63,64].

This Special Issue encompasses diverse research contributions that advance our understanding of fungal infections from diagnostic innovation and clinical assessment, surveillance and risk stratification, therapeutic innovations and drug development, clinical insights and disease mechanisms, epidemiological perspectives and risk factors, through to future directions and clinical implications of AMR and mycology. The multidisciplinary nature of this research, spanning microbiology, immunology, pharmacology, and epidemiology, reflects the complexity of medical mycology as a field.

The collaborative efforts of researchers, clinicians, and public health professionals represented in this collection underscore the global commitment to addressing fungal disease challenges. As we continue to face evolving threats from emerging pathogens and drug resistance, such collaborative research initiatives remain essential for improving patient outcomes and advancing the field of medical mycology. We extend our sincere gratitude to all contributing authors, reviewers, and editorial staff who made this Special Issue possible. Their dedication to advancing fungal infection research will undoubtedly benefit patients and healthcare systems worldwide.
